# ESM1 Interacts with c-Met to Promote Gastric Cancer Peritoneal Metastasis by Inducing Angiogenesis

**DOI:** 10.3390/cancers16010194

**Published:** 2023-12-30

**Authors:** Jiaoyang Yang, Gege Shu, Tao Chen, Anqi Dong, Chao Dong, Weikang Li, Xiaotong Sun, Yajing Zhou, Dongbao Li, Jin Zhou

**Affiliations:** Department of General Surgery, The First Affiliated Hospital of Soochow University, Suzhou 215006, China; jiaoyang0108@163.com (J.Y.); sggshu@163.com (G.S.); 20185232128@stu.suda.edu.cn (T.C.); donganqi@foxmail.com (A.D.); djdwdc@163.com (C.D.); 18021462783@163.com (W.L.); sunxiaotong33@163.com (X.S.); yajingzhou0801@163.com (Y.Z.)

**Keywords:** ESM1, c-Met, gastric cancer, peritoneal metastasis, angiogenesis

## Abstract

**Simple Summary:**

Peritoneum is the most common metastasis site of gastric cancer, and angiogenesis plays a key role in peritoneal metastasis which the specific mechanism of is unknown. We focused on the peritoneal metastasis-associated secretory protein ESM1 based on the previous RNA-seq. The expression of ESM1 is up-regulated in gastric cancer tissues, and even higher in primary gastric cancer with peritoneal metastasis. Moreover, the high expression of ESM1 is significantly associated with poor prognosis. Through clinicopathological data analysis, it was found that ESM1 was positively correlated with vascular invasion. The conditioned medium of gastric cancer cells overexpressing ESM1 promoted tube formation in vitro and gastric cancer cells overexpressing ESM1 induced angiogenesis in vitro. Mechanically, ESM1 binds to c-Met receptor on the surface of endothelial cells, activates the downstream MAPK/ERK signaling pathway, and promotes the transcription and translation of pro-angiogenic molecules (such as HIF1α, VEGFA and MMP9) in endothelial cell, thereby promoting peritoneal metastasis of gastric cancer. Our study may provide a new direction for the diagnosis and treatment of peritoneal metastasis of gastric cancer.

**Abstract:**

The peritoneum is the most common metastatic site of advanced gastric cancer and is associated with extremely poor prognosis. Endothelial-specific molecule 1 (ESM1) was found to be significantly associated with gastric cancer peritoneal metastasis (GCPM); however, the biological functions and molecular mechanisms of ESM1 in regulating GCPM remain unclear. Herein, we demonstrated that ESM1 expression was significantly upregulated in gastric cancer tissues and positively correlated with platelet endothelial cell adhesion molecule-1 (CD31) levels. Moreover, clinical validation, in in vitro and in vivo experiments, confirmed that ESM1 promoted gastric cancer angiogenesis, eventually promoting gastric cancer peritoneal metastasis. Mechanistically, ESM1 promoted tumor angiogenesis by binding to c-Met on the vascular endothelial cell membrane. In addition, our results confirmed that ESM1 upregulated VEGFA, HIF1α, and MMP9 expression and induced angiogenesis by activating the MAPK/ERK pathway. In conclusion, our findings identified the role of ESM1 in gastric cancer angiogenesis and GCPM, thus providing insights into the diagnosis and treatment of advanced gastric cancer.

## 1. Introduction

Gastric cancer (GC) is one of the most common gastrointestinal malignancies worldwide. In 2020, there were 769,000 deaths from gastric cancer among more than one million new cases, ranking it fifth in incidence and fourth in mortality globally [1,2]. Due to the lack of apparent symptoms in the early stage, most patients with gastric cancer present with distant metastasis at the time of diagnosis. The most common site of gastric cancer metastasis is the peritoneum [3], and peritoneal metastasis (PM) is found in about 30% of cases and is usually resistant to systemic chemotherapy [4]. PM has been found to be responsible for the death of about 60% of advanced gastric cancer patients [3]. The median overall survival for PM patients ranges from 6 to 15 months after diagnosis, with only 10% of patients surviving beyond 2 years [5,6]. Therefore, it is urgent to identify the underlying mechanism of PM and the potential therapeutic targets of PM for GC.

According to the “seed and soil” hypothesis, the peritoneal metastasis of gastric cancer depends on the interaction between tumor cells and the peritoneal microenvironment [7]. Peritoneal metastasis is a multistep and disorganized process during which the seeds need to overcome various obstacles, such as ferroptosis [8] and anoikis [9], before they can finally colonize in peritoneum [10]. Many studies have shown that the epithelial–mesenchymal transition (EMT) can promote the peritoneal metastasis of gastric cancer by altering seeds [11]. In addition to this, the formation of new blood vessels after seed colonization is also required to provide subsequent nutrients. Angiogenesis has an important role in tumor metastasis [12]. The newly formed vascular endothelial cells have a disordered arrangement and lose intercellular space [13], which allows the tumor cells to penetrate blood vessels and cause hematogenous dissemination, resulting in the formation of malignant ascites [14,15]. In order to obtain sufficient oxygen and nutrients, tumor cells trigger the release of angiogenic signals, activating the pro-angiogenic signaling pathways and pro-angiogenic factors produced by cancer cells to activate normal, quiescent cells in the metastatic niche, thereby regulating angiogenic events [16,17,18]. Angiogenesis requires the stimulation of vascular endothelial cells through the release of angiogenic factors. The angiogenic process involves the activation, proliferation, and migration of endothelial cells to angiogenic stimuli [19]. Angiogenesis is tightly regulated by the balance between the expression and function of pro-angiogenic and anti-angiogenic mediators. Anti-angiogenic therapy, based on the “starve the tumor to death” premise, has emerged as an attractive strategy to combat various human malignancies, including GC [20,21]. Among the pro-angiogenic factors, the best known and best studied is the vascular endothelial growth factor (VEGF), whose receptor is VEGFR [22]. Anti-angiogenic agents targeting VEGF, such as apatinib, have been approved by the US Food and Drug Administration (FDA) and used in first-line trials for patients with GC [23]. As a result, many studies have shown that ARL13B promotes angiogenesis and tumor growth by activating VEGFA–VEGFR2 signaling in glioma [24]. The ubiquitin E3 ligase SCF (β-TRCP) can promote the ubiquitination and degradation of VEGFR2 in a casein kinase I (CKI)-dependent manner, reduce VEGFR2, and inhibit the angiogenesis and metastasis of thyroid cancer [25].

In our previous study, we performed whole transcriptome sequencing of GC primary and peritoneal metastasis tissues and identified ESM1 as a GCPM-related gene. ESM1, also known as endocan, is a 50-kDa soluble proteoglycan cloned from a human cDNA library in 1996, consisting of a single skin sulfate chain covalently linked to 165 amino acids and with a serine residue at position 137 [26]. It is highly expressed in many kinds of cancers and promotes the occurrence and development of tumors like lung cancer [27], renal clear cell carcinomas [28], gastric cancer [29], and so on. In addition, ESM1 plays an important role in tumor angiogenesis [26]. ESM1 can promote angiogenesis in colorectal cancer (CRC) by activating the PI3K/Akt/mTOR pathway, thereby accelerating tumor progression [30]. The inhibition of ESM1 can reduce angiogenesis and metastasis and attenuate the drug resistance of hepatocellular carcinoma (HCC) [31]. Also, ESM1 has been found to promote angiogenesis in CRC and HCC but not in gastric cancer.

This study found that ESM1 was highly expressed in gastric cancer tissues and positively correlated with microvessel density (MVD). We further demonstrated that ESM1 acted as a secreted protein that significantly promoted the angiogenesis and metastasis of gastric cancer both in in vivo and in vitro by co-culture assays. In addition, our results showed that ESM1 may act as a novel ligand of c-Met and activate the downstream MAPK signaling pathway after binding to c-Met on the surface of endothelial cells to promote the expression of pro-angiogenic molecules, thereby regulating tumor angiogenesis. In conclusion, ESM1 is expected to be a therapeutic target for the peritoneal metastasis of gastric cancer.

## 2. Materials and Methods

### 2.1. Tissue Specimens

Primary gastric cancer tissues and corresponding non-cancerous adjacent tissues were obtained from 180 patients who underwent gastrectomy for gastric cancer in the First Affiliated Hospital of Soochow University. They were followed up, and a clinicopathological and prognostic analysis was performed. The remaining specimens were flash-frozen in liquid nitrogen and then stored in a −80 °C refrigerator for subsequent experiments. All GC patients gave informed consent for the use of clinical specimens for medical research. The Ethics Committee of the First Affiliated Hospital of Soochow University approved this study (approval number: 2022164).

### 2.2. Cell Lines, Cell Cultures, and Transfection

This research used gastric cancer cell lines AGS, HGC27, MKN45, NCI-N87, and SNU-1, bought from Procell Life Science & Technology Co., Ltd. (Wuhan, China); HEK293T and human gastric mucosal epithelial cell line GES-1 were all stored in Dr. Zhou’s laboratory under standard conditions. The HUVEC endothelial cell line was bought from Fu Heng Biology (Shanghai, China). All cell lines were free of mycoplasma contamination. Cells were cultured in RPMI 1640 or ECM medium supplemented with 10% fetal bovine serum (FBS) (Procell, Wuhan, China) and 1% Penicillin/Streptomycin in a cell incubator.

shRNA was purchased from Shandong Virgin Bioscience. The plasmid of ESM1 (with flag label) was purchased from Guan Nan Biotechnology Co., Ltd. (Hangzhou, China). Gastric cancer cells in the logarithmic growth phase were digested with trypsin a day before, resuspended in a complete medium, and counted. An appropriate number of cells were seeded in 6-well plates to ensure that the confluence rate of the cells reached 50% before transfection experiments the next day. After transfection, the 6-well plates were placed in the cell incubator for 48 h. Finally, RT-qPCR and a Western blot were used to detect the overexpression and silencing efficiency of ESM1 mediated by the plasmid and shRNA in gastric cancer cells.

### 2.3. Real-Time Quantitative PCR (RT-qPCR)

The total RNA was extracted and purified using Trizol (Invitrogen, Carlsbad, CA, USA), and then cDNA was synthesized using All-In-One 5 **×** RT MasterMix (ABM, Vancouver, BC, Canada). SYBR Green Master Mix (ABI) was applied to conduct RT-qPCR. The primer sequences are listed in Supplementary Table S1.

### 2.4. Western Blot

Cells were harvested and lysed using RIPA buffer containing 1% PMSF protease inhibitor and phosphatase inhibitor. Protein concentrations were quantified using the BCA Protein Assay kit (Beyotime Biotechnology, Shanghai, China). A certain amount of protein was separated using a jacase preset gel and the proteins were electrotransferred to a PVDF membrane, blocked with skim milk for 2 h, and then incubated with primary antibodies overnight at 4 °C. The next day, the membranes were washed with a TBST solution 3 times/15 min, followed by incubation with the corresponding secondary antibodies for 2 h at room temperature. The membranes were washed again with TBST, 3 times/15 min, after which exposure was performed with ECL (Beyotime Biotechnology, Shanghai, China). Image J/FIJI was used for quantification.

The antibodies against ESM1 (Cat#ab103590), c-Met (Cat#ab216574), p-c-Met (Cat#ab68141), HIF1α (Cat#ab179483), and GAPDH (Cat# ab8245) were purchased from Abcam (Cambridge, MA, USA). The flag antibody (Cat# 20543-1-AP) and C-Myc antibody (Cat#10828-1-AP) were obtained from Proteintech (Wuhan, China). VEGFA (Cat#50661), MMP9 (Cat#13667T), p-RAF (Cat#9421), RAF (Cat#9422), p-MEK (Cat#9154), MEK (Cat#4694), p-ERK1/2 (Cat#4370), ERK1/2 (Cat#9108), p-P38 (Cat#4511), P38 (Cat#9212), p-JNK (Cat#9255), and JNK (Cat#9252) were bought from Cell Signaling Technology (Danvers, MA, USA).

### 2.5. CCK8 Assays

CCK8 assays were performed according to the manufacturer’s instructions (Beyotime Biotechnology, Shanghai, China). One day in advance, 100 μL of cell mixture was added to a 96-well plate and cultured with a conditioned medium. The cells were incubated in the cell incubator for 24, 48, and 72 h. The CCK-8 working solution was added and the absorbance at 450 nm was measured using a microplate reader.

### 2.6. EDU Assay

The CELL-Light EDU Kit was bought from Guangzhou Ruibo Biological Co., Ltd. (Guangzhou, China). The HUVECs in the logarithmic growth phase were seeded in 96-well plates at a rate of 2500 cells per well, after being treated with CM, labeled with EDU, fixed in 4% paraformaldehyde for 30 min at room temperature, and stained with Apollo. They then underwent nuclear staining and were finally observed by laser confocal microscopy.

### 2.7. Wound Healing Assay

The logarithmic growth of HUVEC cells was also investigated. They were seeded in a 6-well plate, and, after reaching 90% confluence, a wound was drawn on the bottom of the 6-well plate with a 100 µL pipet tip, and a different CM was added to continue the culture for 24 h. The wound healing rate was calculated by microscope.

### 2.8. Cell Migration and Invasion Assays

Cell migration and invasion assays were performed using 24-well plates. The chamber was lined with pre-treated matrigel the day before the experiments were performed, and GC cells were digested and resuspended in ECM medium the next day and then counted. A total of 200 μL (5 × 10^5^ cells/mL) of GC cells were added to the chamber, and 600 μL of ECM was added to the lower chamber. The cells were incubated in a cell incubator at 37 °C for 36 h. After reaching the incubation time, the cells were washed twice with PBS, fixed with 4% paraformaldehyde for 20 min, washed twice with PBS, stained with crystal violet for 1 h, washed twice with PBS, and then the matrigel at the bottom of the chamber was scraped off with a cotton swab. The invading cells were counted in five random fields at 400× magnification. Each experiment was repeated three times, and its mean value was calculated.

In contrast to the invasion assay, the matrigel paving was omitted from the migration assay, and the other procedures were consistent. In total, 200 μL (5 × 10^4^ cells/mL) of GC cells were evenly spread in the chamber, and 600 μL of ECM was added to the 24-well plate. The cells were incubated for 12 h in a cell incubator. After the incubation time was reached, the invaded cells were counted in five random fields at ×400 magnification after washing the fixed stain. Each experiment was repeated three times, and its mean value was calculated.

### 2.9. Preparation of Conditioned Medium

Gastric cancer cells in the logarithmic growth phase were collected, and the cell concentration was adjusted to 2 × 10^6^ cells/mL. The cells were cultured in ECM, penicillin 100 U/mL, and streptomycin 100 mg/mL for 48 h; then, the original medium was discarded. The gastric cancer cell culture supernatant was collected the next day, centrifuged at 2000 r/min for 5 min, filtered through a microporous filter with a diameter of 0.22 μm to remove floating tumor cells, and prepared at a volume ratio of 50%.

### 2.10. Tube Formation Assay

The HUVEC cells were cultured in ECM for 24 h the day before the experiment. The next day, HUVECs in the logarithmic growth phase were resuspended by trypsin digestion and counted, and 250 µL of matrigel was spread in precooled 24-well plates and placed in a cell incubator for 30 min of incubation to solidify the matrigel. When 300 µL of medium was mixed with 10 µL of cell suspension spread in a 24-well plate, tubules would form within 2–4 h. After 12 h, the medium was removed and fixed with 4% paraformaldehyde for 15 min at room temperature before being photographed.

### 2.11. In Vivo Chick Chorioallantoic Membrane (CAM) Angiogenesis Assays

Eggs with embryos were incubated at 37 °C in an incubator with 70% humidity. After 6 to 7 days of incubation, an air chamber was made, the shell membrane and chorioallantoic membrane were separated, and a window was opened on the shell membrane for injection. The CM from GC-shESM1 cells with or without recombinant ESM1, MAPK/ERK inhibitor SCH772984 (Selleck, Houston, TX, USA) or c-Met-inhibitor AMG-337 (Shanghai Ulva test), and the CM from GC cells with or without the ESM1-neutralizing antibody and MAPK/ERK stimulator Ceramide C6 (Santa Cruz, CA, USA) or MAPK/ERK inhibitor SCH772984 (Selleck, USA) were added to the loop by window injection. After 3 days, the CAM area was isolated, unfolded on a glass slide, and imaged under a stereomicroscope. Vascularization was assessed using image J/FIJI.

### 2.12. In Vivo Matrigel Plug Assay

Matrigel (BD Biosciences, #354262) was mixed with 100 ng/mL of ESM1 or CM with AGS -shNC/shESM1 cells or HGC27-vector/ESM1 cells at a ratio of 2:3 (total volume 1 mL) and then subcutaneously injected into the right side of C57 female mice aged 5–6 weeks. There were three animals in each group. The plug was taken 7 days after injection and the hemoglobin content was determined by the Drabkin method.

### 2.13. Immunoprecipitation Assays

A total of 5 mg of total cell lysate was immunoprecipitated overnight at 4 °C with anti-Flag or anti-Myc beads (Beyotime, China). Extensive washing and immune complex denaturation steps were performed according to the manufacturer’s instructions. The denatured immune complexes were analyzed by immunoblotting. IgG was used as a negative control. Western blot analysis was performed using standard protocols.

### 2.14. GST Pulldown Assay

The expression of the pGEX-4T-1 empty vector and recombinant ESM1 expressing vectors in BL21 host *E. coli* were induced by 0.2 mM IPTG at 21 °C for 16 h. GST and GST-ESM1 were purified using GST agarose beads, followed by overnight incubation with the cell lysates of HUVECs (overexpressing Myc-c-Met) in a rolling incubator at 4 °C. The next day, after washing three times with PBS, SDS loading buffer was added into the beads and boiled for 5 min for subsequent Western blot analysis using antibodies against Myc.

### 2.15. Immunohistochemistry

Clinical and mouse specimens were subjected to immunohistochemical staining using 5 μm thick paraffin-embedded sections. The sections were dewaxed before the antigen repair, and the specimens were blocked with 10% sheep serum at room temperature for 1 h. After the blocking agent was removed, the tissue slices were framed with water-blocking pens, and 50 µL of primary antibody was dropped on each tissue, placed at 37 °C for 1 h, and washed four times with PBS for 5 min each time. Secondary antibodies were incubated at 37 °C for 30 min, washed with PBS, and freshly prepared with DAB color solution; 50 µL was added to each tissue to control the color development under the microscope, and the color development was terminated with PBS. The plates were counterstained with hematoxylin, rinsed with water for 20–30 min, dehydrated, sealed, and photographed.

### 2.16. Xenograft Assay and Bioluminescence Imaging

Male BALB/c nude mice, aged 4 weeks, were purchased from Shanghai SLAC Laboratory Animal Co., Ltd. (Shanghai, China) and all mice were about the same size and weight. The purchased nude mice were kept in SPF-class housing in the laboratory. All animal studies (including mouse euthanasia procedures) were performed in compliance with Soochow University Institutional Animal Care Regulations and in accordance with AAALAC and IACUC guidelines (approval number: 202209A0101).

The nude mice were fed for about a week before the model was constructed. To establish a subcutaneous tumor model, AGS cells were digested with trypsin, resuspended in sterile PBS, and counted so that there were 1 × 10^7^ cells in 200 µL of the cell suspension. A total of 200 µL of the cell suspension was extracted from a 1 mL syringe and injected into the abdominal cavity of nude mice (five mice per group). Sterile PBS/ESM1 Ab/SCH772984 was injected via the tail vein on days 3, 6, 9, 12, 15, 18, and 21; the xenograft animal model was analyzed using IVIS Spectrum at the 7, 14, and 21 day time points.

### 2.17. Statistical Analysis

Statistical analysis was performed using SPSS (version 16.0). A paired Student t-test, independent Student t-test, and chi-squared test were used for data statistics. Survival curves were generated using the Kaplan–Meier method. Univariable and multivariable Cox proportional hazard regression models were used to analyze independent prognostic factors. The data are presented as the mean SD of three independent experiments. A *p*-value less than 0.05 was considered statistically significant.

## 3. Results

### 3.1. ESM1 Is Upregulated in Primary and Metastatic GC Tissues and Positively Associated with Tumor Angiogenesis in GC

**Figure 1:**
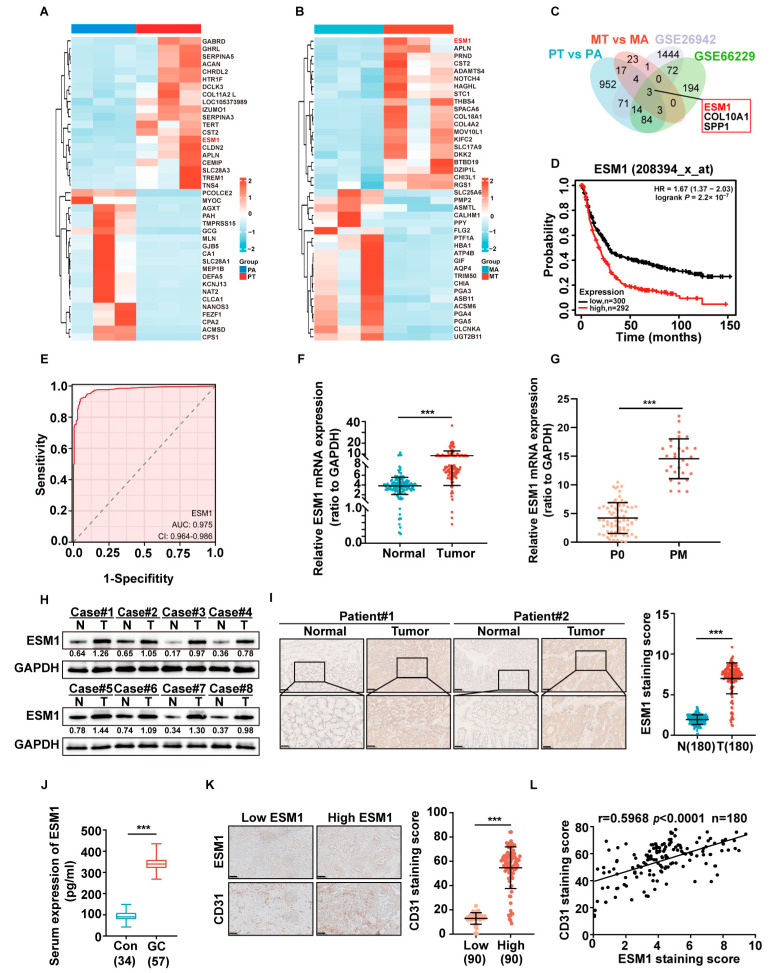
ESM1 is up-regulated in primary and metastatic GC tissues and is positively correlated with tumor angiogenesis in GC. The heatmap mainly indicates the expression levels of 20 differential up-regulated and down-regulated genes in primary gastric cancer (**A**) and metastatic gastric cancer (**B**), relative to adjacent tissues. (**C**) A Venn plot represents the three overlapped up-regulated genes. (**D**) **A** KaplanMeier plot of OS for GC samples in the KM plot database. (**E**) ROC curve analysis of the sensitivity and specificity of ESM1 in the diagnosis of gastric cancer patients (AUC = 0.975, *p* < 0.05). (**F**) RT-qPCR analysis of ESM1 mRNA expression in 77 pairs of gastric cancer patient specimens. Data are presented as the mean ± SD of triplicate independent sets of experiments. Statistical significance was assessed by a paired *t*-test, *** < 0.001, *n* = 77. (**G**) RT-qPCR analysis of ESM1 mRNA expression in 71 gastric cancer patient specimens without PM and 30 gastric cancer patient specimens with PM. Data are presented as the mean ± SD of triplicate independent sets of experiments. Statistical significance was assessed by a paired *t*-test, *** < 0.001, *n* = 71. (**H**) A Western blot analysis was performed on eight pairs of GC patient samples using an antibody against ESM1. The uncropped blots are shown in Supplementary Materials Figure S5C. (**I**) IHC staining was performed using an antibody against ESM1 and representative photographs of ESM1 in 180 GC patients. Scale bar: 100 μm. The right is the IHC stain scoring of ESM1. Statistical significance was assessed by an unpaired *t*-test, *** < 0.001. (**J**) A Serum ELISA assay of 34 normal people and 57 GC patients revealed that ESM1 was highly expressed in GC patients’ serum compared with normal people. Con: control group; GC: gastric cancer patients. Statistical significance was assessed by an unpaired *t*-test, *** < 0.001. (**K**) IHC staining was performed using antibodies against ESM1 and CD31, and representative photographs of CD31 in 180 GC tissues with a high and low ESM1 expression. Scale bar: 100 μm. The right includes the IHC staining scores of CD31. Statistical significance was assessed by a paired *t*-test, *** < 0.001. (**L**) A correlation analysis of the staining index of the protein expression levels of ESM1 and CD31 in GC specimens (*n* = 180). The correlation coefficient is shown. The data represent the means ± SEM, *** < 0.001.

In order to find novel genes associated with GC peritoneal metastasis, we compared their gene expression in three pairs of primary GC tissues (PT) and adjacent tissues (PA), as well as three paired peritoneal metastasized GC tissues (MT and MA) by RNA-seq; we then selected 1148 (PT vs. PA) and 51 (MT vs. MA) differential genes, respectively, for subsequent analysis. The heat map shows genes with significantly differential expression in PT vs. PA and MT vs. MA (Figure 1A,B). ESM1 expression significantly differed, and the volcano map showed consistent results (Figure S1A,B). Next, we Venn diagrammed the upregulated genes of the above four gene sets for overlapped genes, which showed three screened genes (Figure 1C). GEO datasets (GSE26942 and GSE66229) and the TCGA database further confirmed that ESM1 was highly expressed in gastric cancer tissues (Figure S1C–E). In addition, we found that GC patients with higher ESM1 levels had a lower overall survival using the Kaplan–Meier Plotter database (Figure 1D). In addition, ESM1 greatly improved the sensitivity and specificity of the gastric cancer diagnosis (Figure 1E).

To further examine the expression level of ESM1 in gastric cancer tissues, we detected 77 paired adjacent and GC tissues by RT-qPCR, and the results showed that ESM1 expression was higher in gastric cancer tissues than in adjacent tissues (Figure 1F). We also examined ESM1 expression in 71 primary cancer tissues without PM and 30 primary cancer tissues with PM and found that primary tissues with PM had a higher ESM1 expression (Figure 1G). The Western blot yielded results consistent with RT-qPCR (Figure 1H). The same results were obtained by immunohistochemistry (Figure 1I). Moreover, ESM1 was highly expressed in the serum of GC patients (Figure 1J).

The clinical data sheet suggested that high ESM1 expression was closely related to vascular invasion (Table S2), so we detected the expression of angiogenesis targets (CD31 and VEGFA) in adjacent and GC tissues by Western blot, and the results showed that CD31 and VEGFA expression was higher in gastric cancer tissues than in adjacent tissues (Figure S1F,G). We also performed platelet endothelial cell adhesion molecule-1 (CD31, which is often used as a marker for angiogenesis) staining on tumor tissues with high or low ESM1 expression, finding that tumor tissues with a high ESM1 expression had higher CD31 expression (Figure 1K). In addition, a correlation analysis showed that ESM1 expression was positively correlated with CD31 (Figure 1L).

### 3.2. Tumor Cell-Derived ESM1 and Recombinant ESM1 Induce Specifically Endothelial Cell Responses

Next, we examined the expression levels of ESM1 in GC cell lines which were higher than in human gastric epithelial cell GES-1 (Figure S2A,B). AGS and HGC27, with the highest and lowest expression of ESM1, were selected for subsequent experiments. Given that ESM1 is a secreted protein, we measured the amount of ESM1 in the culture supernatant of each cell line by ELISA (Figure S2C). Subsequently, the HGC27 cell was used to stably overexpress ESM1 and AGS cells to knock down ESM1, and the transfection efficiency was verified by RT-qPCR and Western blot. The level of ESM1 in the supernatant was also increased or decreased (Figure S2D–I).

**Figure 2:**
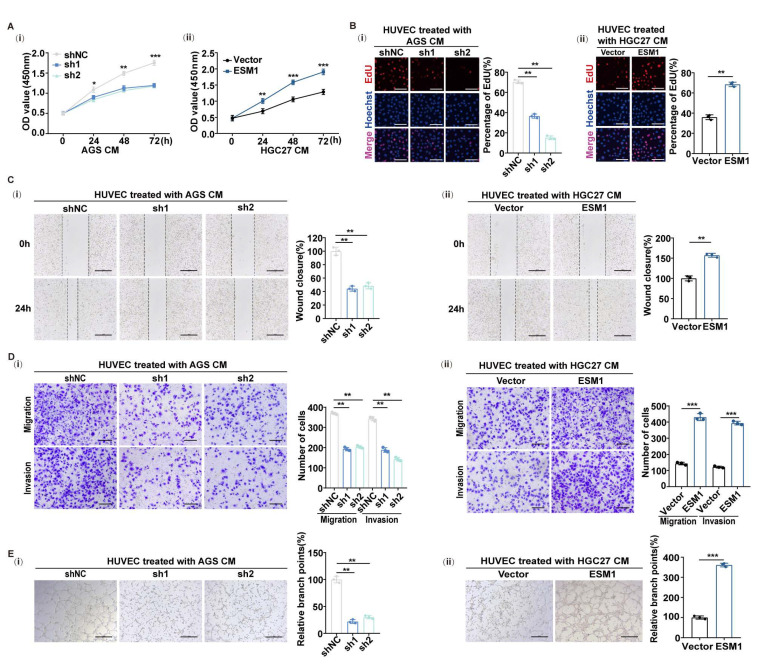
Tumor cell-derived ESM1 and recombinant ESM1 induce specifically endothelial cell responses. (**A**) (**i**) A CCK8 assay analyzed the proliferation of HUVECs treated with the CM of AGS-shNC/AGS-shESM1-1/AGS-shESM1-2 and (**ii**) HGC27-Vector/HGC27-ESM1 stable cell lines. Data are shown as the mean ± SD of triplicate independent sets of experiments; statistical significance was assessed by a paired *t*-test, * < 0.05, ** < 0.01, and *** < 0.001. (**B**) (**i**) An EDU assay analyzed the proliferation of HUVECs treated with the CM of AGS-shNC/AGS-shESM1-1/AGS-shESM1-2 and (**ii**) HGC27-Vector/HGC27-ESM1 stable cell lines. Representative images (**left panel**) and quantification (**right panel**) are shown as indicated. Data from independent experiments are presented as the mean ± SD. Statistical significance was assessed by an unpaired *t*-test. ** < 0.01. Scale bar: 50 μm. (**C**) (**i**) A wound healing assay analyzed the migration of HUVECs treated with the CM of AGS-shNC/AGS-shESM1-1/AGS-shESM1-2 and (**ii**) HGC27-Vector/HGC27-ESM1 at 0 and 24 h. Representative images (**left panel**) and quantification (**right panel**) are shown as indicated. Data from independent experiments are presented as the mean ± SD. Statistical significance was assessed by an unpaired *t*-test. ** < 0.01. Scale bar: 100 μm. (**D**) Transwell migration and Matrigel invasion assays were performed to assess the migration and invasion ability of HUVECs treated with the CM of (**i**) AGS-shESM1-1/AGS-shESM1-2 and (**ii**) HGC27-vector/HGC27-ESM1. Representative images (**left panel**) and quantification (**right panel**) are shown as indicated. Data from independent experiments are presented as the mean ± SD. Statistical significance was assessed by an unpaired *t*-test. ** < 0.01, *** < 0.001. Scale bar: 50 μm. (**E**) A tube formation assay was performed to assess the tube-forming ability of HUVECs treated with the CM of (**i**) AGS-shESM1-1/AGS-shESM1-2 and (**ii**) HGC27-vector/HGC27-ESM1. Representative images (**left panel**) and quantification (**right panel**) are shown as indicated. Data from independent experiments are presented as the mean ± SD. Statistical significance was assessed by an unpaired *t*-test. ** < 0.01, *** < 0.001. Scale bar: 200 μm.

To investigate the effect of ESM1 secreted by gastric cancer cells on endothelial cells, we treated endothelial cells with gastric cancer cell culture supernatant to detect the effect on their functional behaviors. The CCK8 and EDU array showed that the proliferation ability of HUVECs treated with the supernatant of AGS-shESM1-1 and AGS-shESM1-2 could be inhibited compared with the control cell supernatant (Figure 2(A-i,B-i)), while the proliferation of HUVECs treated with the supernatant of HGC27-ESM1 could be enhanced compared with the control group (Figure 2(A-ii,B-ii)). Moreover, the wound healing assay showed that the migratory ability of HUVECs was inhibited when treated with the supernatant of AGS-shESM1-1 or AGS-shESM1-2 (Figure 2(C-i)). When treated with the supernatant of HGC27-ESM1, the migratory ability was increased (Figure 2(C-ii)). A Transwell assay further revealed that HUVECs migration and invasion slowed down when treated with the supernatant of AGS-shESM1-1 and AGS-shESM1-2 (Figure 2(D-i)), while they increased when treated with the supernatant of HGC27-ESM1 (Figure 2(D-ii)). The tube formation assay showed that ESM1 could promote tube formation. HUVECs treated with AGS-shESM1-1/AGS-shESM1-2 cell supernatant showed less tube formation (Figure 2(E-i)), while HUVECs treated with HGC27-ESM1 cell supernatant showed more tube formation compared with the negative control group (Figure 2(E-ii)). The cell supernatant is a collection of cytokines; therefore, in order to clarify the function of ESM1, we added recombinant ESM1 to the supernatant of the HGC27-vector or added neutralizing antibody against ESM1 to the supernatant of AGS-shNC and then treated the HUVECs. All these cellular functions could be rescued, as shown in Figure S3A–E.

### 3.3. Tumor Cell-Derived ESM1 Promotes GC Metastasis and Angiogenesis

**Figure 3:**
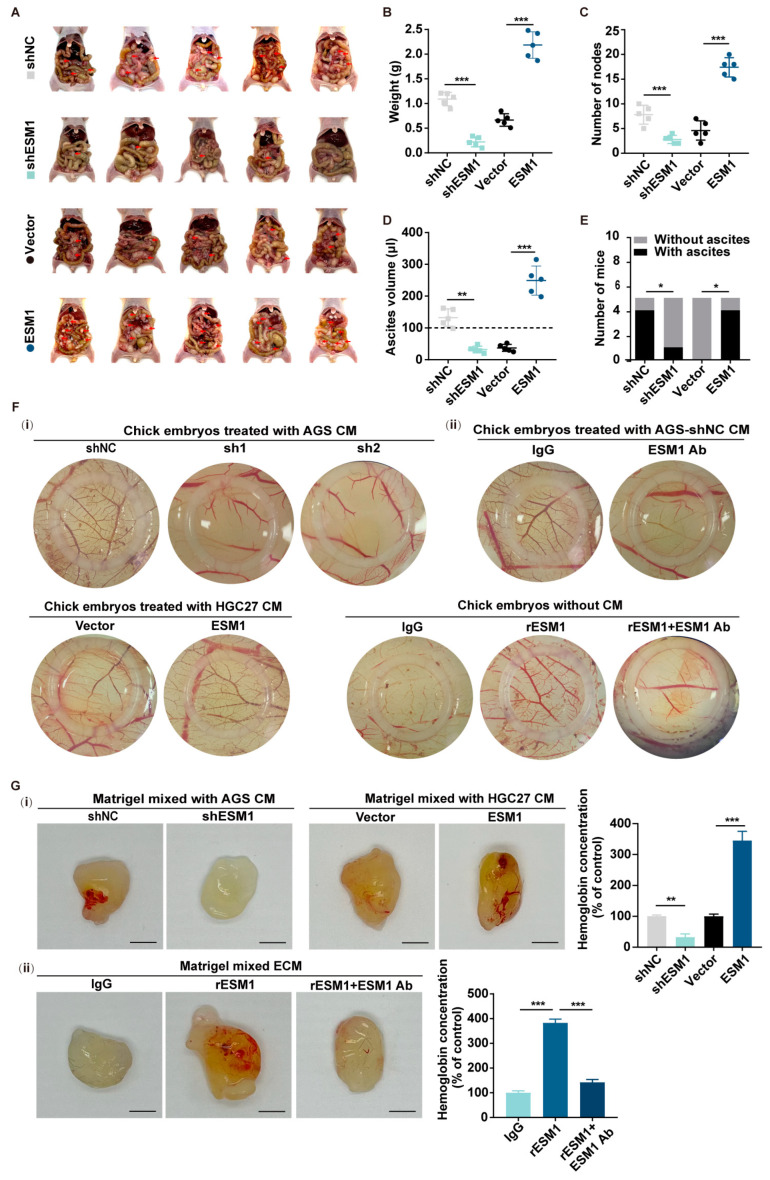
Tumor cell-derived ESM1 promotes angiogenesis and GC metastasis. (**A**) Images of peritoneal metastasis in nude BALB/c mice after injection. Arrows point to typical examples. (**B**) Statistical significance of the peritoneal nodes’ weights. *** < 0.001. (**C**) Number of peritoneal metastases obtained from mice in each group. *** < 0.001. (**D**) Ascites volume of mice in each group. ** < 0.01, *** < 0.001. (**E**) Number of mice with or without ascitic fluid in each group. * < 0.05. (**F**) (**i**) A CAM assay was performed to assess angiogenic ability after being treated with the CM of AGS-shESM1-1/AGS-shESM1-2 and HGC27-vector/HGC27-ESM1. (**ii**) A CAM assay was performed to assess angiogenic ability after being treated with CM mixed with recombinant ESM1 or neutralizing antibody against ESM1 and the CM of AGS-shNC mixed with a neutralizing antibody against ESM1. The inner diameter of the sampling rings was 8 mm. (**G**) A Matrigel Plug assay was performed to assess angiogenic ability by post-subcutaneous injection of matrigel mixed with either shNC CM or shESM1 CM from AGS stable cells or the vector CM or ESM1 CM from HGC27 stable cells (**i**) or recombinant ESM1 (100 ng/mL) or ESM1 Ab or IgG (negative control) (**ii**). Representative images (**left**) and hemoglobin quantification (**right**). Data from independent experiments are presented as the mean ± SD. Statistical significance was assessed by an unpaired *t*-test. ** < 0.01, *** < 0.001.

We next investigated the role of ESM1 in gastric cancer metastasis. First, we performed KEGG and GSEA enrichment analysis and found that angiogenesis was highly enriched in the ESM1 high expression group in PT vs. PA and MT vs. MA (Figure S4A,B). We injected AGS-shNC, AGS-shESM1 cells, and HGC27-Vector as well as HGC27-ESM1 cells into the peritoneal cavity of nude mice. After 3 weeks, the peritoneal metastasis was analyzed. As expected, knocking down ESM1 significantly decreased the metastatic progression (Figure 3A), tumor weight (Figure 3B), number of disseminated tumor nodes (Figure 3C), ascites volume (Figure 3D), and number of mice with ascites (Figure 3E), while overexpressing ESM1 increased the above indicators. The chick chorioallantois membrane assay showed that ESM1 could promote angiogenesis. The membrane treated with AGS-shESM1-1/AGS-shESM1-2 cell supernatant showed fewer blood vessels, while that treated with HGC27-ESM1 cell supernatant showed more angiogenesis compared with the control group (Figure 3(F-i)). The chick embryos were then treated with a medium containing recombinant ESM1 or a neutralizing antibody against ESM1, and IgG was used as a control. It was found that the angiogenesis ability was enhanced after the addition of recombinant ESM1, but it weakened again after the addition of neutralizing antibodies against ESM1. In addition, the embryos were treated with an AGS-shNC cell medium supplemented with neutralizing antibodies against ESM1, and IgG was used as the control group. The blood vessels were seen to be reduced (Figure 3(F-ii)). To further evaluate the contribution of ESM1 to the tube-forming phenotype development of HUVECs, we examined the impact of ESM1 in an in vivo Matrigel plug assay, and the results were consistent with the chick chorioallantois membrane assay (Figure 3(G-i,ii)).

### 3.4. ESM1 Interacts with Membrane Receptor c-Met and Promotes Angiogenic Factor Expressions

**Figure 4:**
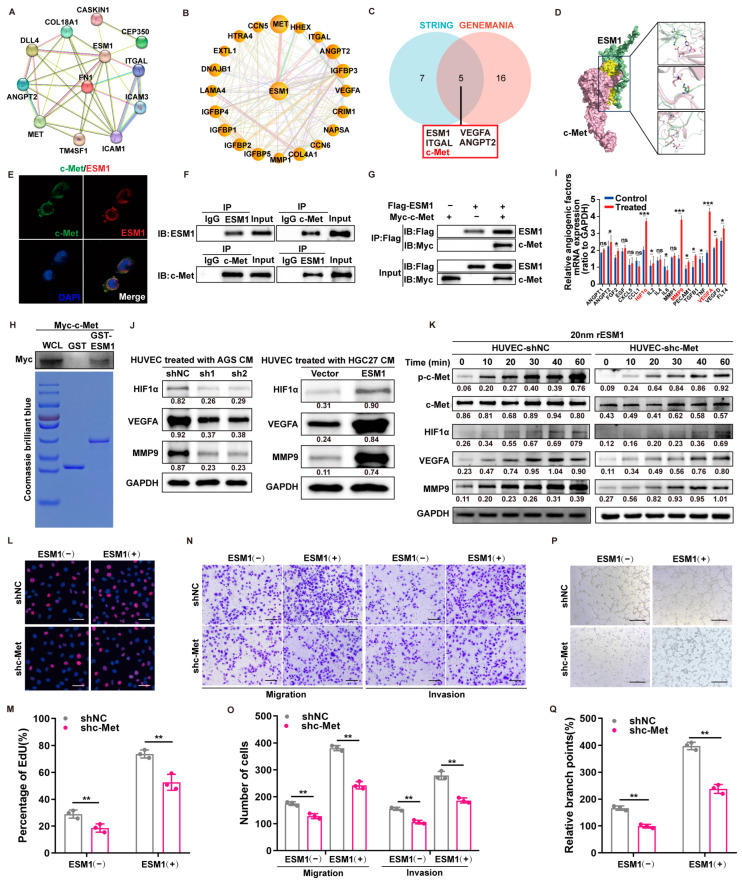
ESM1 interacts with membrane receptor c-Met and promotes pro-angiogenic factor expression. Predicted proteins could interact with ESM1 based on the STRING (**A**) and GENEMANIA (**B**) database. (**C**) The Venn showed overlapped proteins. (**D**) The binding complex models of ESM1 and c-Met were predicted by using the Cluspro online protein docking tool. (**E**) HUVECs were treated with recombinant ESM1 for 2 h, followed by double immunofluorescence staining with c-Met (green) ESM1 (red) antibodies. Nuclei were counterstained with DAPI (blue). (**F**) The immunoprecipitation of the ESM1 protein by an anti-c-Met antibody and the immunoprecipitation of the c-Met protein by an anti-ESM1 antibody. IgG was used as a negative control. The uncropped blots are shown in Supplementary Materials Figure S5D. (**G**) Immunoprecipitation of the c-Met protein by an anti-Flag antibody in HUVECs (transfected with Myc-c-Met) treated with the CM of HEK293 cells transfected with Flag-ESM1. The uncropped blots are shown in Supplementary Materials Figure S5D. (**H**) GST pulldown was performed to determine the direct interaction between ESM1 and c-Met. Recombinant GST and GST-ESM1 were purified from bacteria and analyzed by SDS-PAGE and Coomassie blue staining. (**I**) The fold change of classic pro-angiogenic factor expression in HUVECs treated with recombinant ESM1. ns: no significance; * < 0.05 and *** < 0.001. (**J**) The Western blot analyzed pro-angiogenic factors showed protein expression in HUVECs treated with the CM of AGS-shNC/AGS-shESM1-1/AGS-shESM1-2 and HGC27-Vector/HGC27-ESM1. The uncropped blots are shown in Supplementary Materials Figure S5D. (**K**) The Western blot analysis of lysates from HUVECs starved for 6 h and then incubated with recombinant ESM1 for up to 60 min, followed by the immunoblotting of lysates The uncropped blots are shown in Supplementary Materials Figure S5D. (**L**) The EDU assay and its quantification (**M**) are shown as indicated. Data from independent experiments are presented as the mean ± SD. Statistical significance was assessed by an unpaired *t*-test. ** < 0.01. Scale bar: 50 μm. (**N**) The Transwell assay and its quantification (**O**) are shown as indicated. Data from independent experiments are presented as the mean ± SD. Statistical significance was assessed by an unpaired *t*-test. ** < 0.01. Scale bar: 50 μm. and (**P**) Tube formation and its quantification (**Q**) are shown as indicated. Data from independent experiments are presented as the mean ± SD. Statistical significance was assessed by an unpaired *t*-test. ** < 0.01. Scale bar: 50 μm. All experiments were performed in the absence or presence of recombinant ESM1. Graph of the average number of meshes as described in Section 2.

In order to study the mechanism of ESM1-promoting GC metastasis, we first predicted the possible binding proteins of ESM1 by two protein interaction prediction databases, STRING (Figure 4A) and GENENAMIA (Figure 4B), and then obtained five overlapped proteins, including c-Met (Figure 4C). Furthermore, protein–protein docking was used to predict the complex 3D model and potential interaction domains of ESM1 and c-Met (Figure 4D). We then treated HUVECs with recombinant ESM1 and confirmed that ESM1 as well as c-Met were colocalized at the cell membrane by immunofluorescence (Figure 4E). To further demonstrate the protein–protein interaction between ESM1 and c-Met, co-IP was performed after HUVECs were treated with the supernatant of AGS overexpressed ESM1, and the results showed an interaction between two proteins (Figure 4F). Next, the HEK293T cells were transiently transfected with Flag-tagged ESM1 expression plasmid, and the HUVECs were transiently transfected with the Myc-tagged c-Met expression plasmid, followed by treatment of the latter with the former supernatant, and, finally, co-IP with anti-Flag and anti-Myc antibodies (Figure 4G). Moreover, in order to test whether the two proteins interact directly, we performed a GST pull-down assay, and the result showed that EMS1 and c-Met interact directly (Figure 4H). These results indicated that ESM1 could bind to c-Met. Next, the expression of classical pro-angiogenic factors in recombinant ESM1-treated HUVECs was examined by RT-qPCR, revealing that HIF1α, VEGFA, and MMP9 were upregulated (Figure 4I); Western blot further confirmed this finding (Figure 4J).

We also measured the phosphorylation level of c-Met and the expression of pro-angiogenic molecules by treating HUVEC-shNC and HUVEC-shc-Met with recombinant ESM1 at 0, 10, 20, 30, 40, and 60 min, respectively. Our results showed that protein expression increased with the extension of the treatment time. The relative protein expression decreased again after knocking down the c-Met in HUVECs (Figure 4K). We knocked down c-Met in the HUVECs for the rescue experiment, followed by treatment with or without recombinant ESM1. The EDU assay showed that the addition of recombinant ESM1 successfully rescued the reduced proliferation caused by c-Met being knocked down (Figure 4L), and quantification (Figure 4M) are shown as indicated. The migration and invasion assay results showed that the c-Met deficiency largely abolished the promotion effect of ESM1 on HUVECs (Figure 4N), and quantification (Figure 4O) are shown as indicated. Similarly, the tube formation assay showed that recombinant ESM1 successfully rescued the reduced tube formation ability caused by c-Met being knocked down (Figure 4P), and quantification (Figure 4Q) are shown as indicated.

### 3.5. ESM1 Promotes Angiogenesis through MAPK/ERK Signaling Pathway

**Figure 5:**
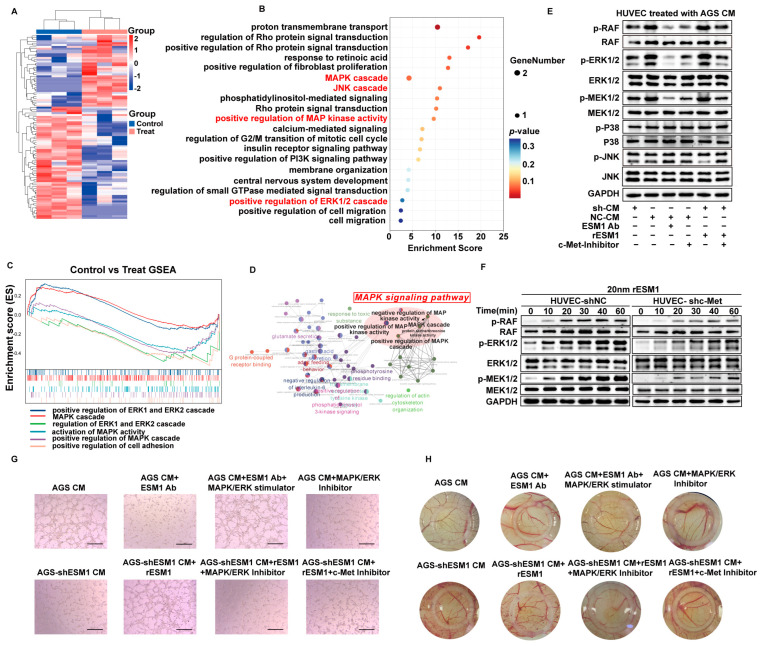
ESM1 promotes angiogenesis through the MAPK/ERK signaling pathway. (**A**–**D**) RNA sequencing (RNA-seq) was performed on HUVECs treated with the CM obtained from GC cells in the presence or absence of a neutralizing antibody against ESM1. (**A**) Heatmap of significantly differential genes. (**B**,**C**) The gene set enrichment analysis of gene ontology performed using the cluster Profiler R package based on differential genes. (**B**). Bubble chart of the top 20 enriched GO biological processes processed using ggplot2 R package. (**C**) Enrichment map of specific GO sets of interest. (**D**) GO enrichment network analysis performed with cluego, a Cytoscape plug-in based on significantly differential genes. (**E**) The protein expression of HUVECs treated with the CM obtained from AGS-shESM1 cells in the presence or absence of recombinant ESM1 or c-Met-inhibitor; AGS cells in the presence or absence of a neutralizing antibody against ESM1 or the c-Met-inhibitor detected using Western blot with indicated antibodies. The uncropped blots are shown in Supplementary Materials Figure S5E. (**F**) The protein expression in HUVEC-shNC and HUVEC-shc-Met cells treated with recombinant ESM1 at 0, 10, 20, 30, 40, and 60 min detected using a Western blot with indicated antibodies. The uncropped blots are shown in Supplementary Materials Figure S5E. (**G**) The Tube formation of HUVECs treated with the CM obtained from AGS-shESM1 cells in the presence or absence of recombinant ESM1, MAPK/ERK inhibitor or c-Met-inhibitor, AGS cells in the presence or absence of neutralizing antibody against ESM1, MAPK/ERK stimulator, or MAPK/ERK-inhibitor Scale bar: 200 µm. (**H**) The results of the CAM assay performed using embryonated chicken eggs treated with the CM obtained from AGS-shESM1 cells in the presence or absence of recombinant ESM1, MAPK/ERK inhibitor, or c-Met-inhibitor, AGS cells in the presence or absence of neutralizing antibody against ESM1, MAPK/ERK stimulator or MAPK/ERK-inhibitor. The inner diameter of the sampling rings was 8 mm.

In order to explore how ESM1 promotes HUVEC tube formation and to determine the major downstream signaling events of ESM1 in HUVECs, RNA-seq was performed on HUVECs treated with AGS CM in the presence or absence of a neutralizing antibody against ESM1. The heat map showed differential genes in groups with or without ESM1-neutralizing antibodies (Figure 5A). A GO and GSEA enrichment analysis showed that the MAPK pathway was significantly enriched (Figure 5B,C). Furthermore, a GO enrichment network analysis using significantly differentially expressed genes identified MAPK signaling as the main and most dominant upstream regulator of the GC-derived ESM1-triggered gene ontology network, indicating the possible central role of MAPK signaling in ESM1-mediated angiogenesis (Figure 5D). The MAPK cascade consists of three branches: p38, JNK, and ERK, among which only the MAPK/ERK cascade was clearly activated in CM-treated HUVECs, as determined by the phosphorylation of MAPKKK/Raf, MAPKK/MEK1/2, and MAPK/ERK1/2 (Figure 5E). Neutralizing antibodies against ESM1 and the inhibition of c-Met could reverse the elevated activation of HUVECs treated by GC cells’ CM. Furthermore, supplementation with recombinant ESM1 could activate the MAPK/ERK cascade but not the MAPK/P38 or MAPK/JNK cascades. In addition, the ESM1-induced increase in HUVEC activation was significantly attenuated by the inhibition of c-Met, the major receptor of ESM1. The above results suggest that GC-derived ESM1 can activate the MAPK/ERK cascade via c-Met. When HUVEC-shNC or HUVEC-shc-Met were treated with rESM1 for 0, 10, 20, 30, 40, and 60 min, the phosphorylation of the MAPK/ERK pathway was increased with the extension of treatment time. In addition, the phosphorylation of MAPK/ERK was decreased after knocking down c-Met in HUVECs (Figure 5F). Furthermore, the inhibition of MAPK/ERK or the ESM1 receptor blocked the pro-angiogenic effects induced by recombinant ESM1 in vitro (Figure 5G) and in vivo (Figure 5H). Stimulation of the MAPK/ERK cascade rescued the anti-angiogenic effect induced by neutralizing the antibodies against ESM1 in vitro and in vivo. The above results suggest that GC cell-derived ESM1 promotes HUVEC angiogenesis through c-Met-mediated MAPK/ERK cascade activation.

### 3.6. ESM1/c-Met Axis Triggers Angiogenesis and Peritoneal Metastasis In Vivo

**Figure 6:**
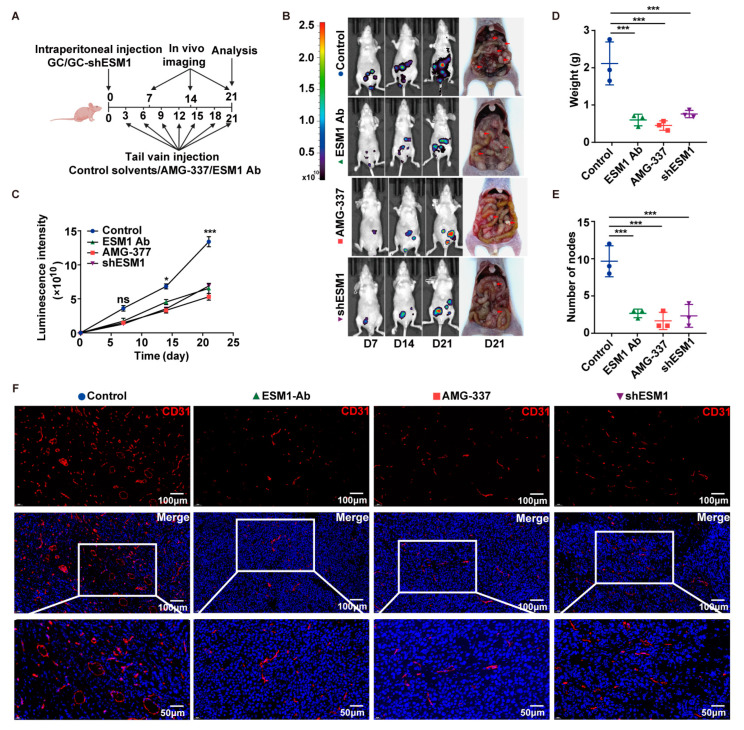
The ESM1/c-Met axis triggers angiogenesis and peritoneal metastasis in vivo. (**A**–**C**) The peritoneal metastasis model was established by intraperitoneal injection with GC cells with GC-shESM1 or GC cells, followed by targeted treatment using a neutralizing antibody against ESM1, c-Met specific inhibitor AMG-337, or control saline, and bioluminescence imaging. (**A**) The detailed flow chart. (**B**) Bioluminescence imaging was performed using IVIS Lumina XR at weeks 1, 2, and 3. (**C**) The average luminescence intensity of mice in each group at the indicated time. ns: no significance, * < 0.05 and *** < 0.001. (**D**) The weight of peritoneal metastases obtained from mice in each group. *** < 0.001. (**E**) The number of peritoneal metastases obtained from mice in each group. *** < 0.001. (**F**) Representative images of the immunofluorescence staining of CD31 in the peritoneal metastases of mice in each group.

In order to investigate whether the effect of angiogenesis in vitro could be recapitulated in vivo, peritoneal metastasis models were established by the intraperitoneal injection of GC cells or GC-shESM1 cells, followed by targeted therapy and bioluminescence imaging. Figure 6A shows the detailed flow diagram. Treatment with a neutralizing antibody against ESM1, c-Met specific inhibitor-AMG-337, and the knocking down of ESM1 significantly inhibited metastatic progression (Figure 6B,C), the weight of nodes (Figure 6D), and the number of nodes (Figure 6E). In addition, treatment with neutralizing antibodies against ESM1, AMG-337, or knocking down ESM1 significantly inhibited angiogenesis, as characterized by CD31 staining and the reduced MVD in the peritoneal metastases of mice (Figure 6F). These data reveal the important role of ESM1 and c-Met in metastatic angiogenesis.

## 4. Discussion

As a common malignant solid tumor, gastric cancer is more prone to peritoneal metastasis [3]. Angiogenesis is one of the characteristics of gastric cancer cells, and the biological basis as well as a critical factor for malignant transformation, tumor growth, and metastasis [12]. Due to the evolutionary development of cancer cells, their resistance to targeted drugs gradually grows. Therefore, exploring the potential mechanism of gastric cancer peritoneal metastasis and new therapeutic targets for peritoneal metastasis is of utmost importance. Herein, we reported, for the first time, that ESM1 activates the downstream MAPK pathway by binding to the membrane receptor c-Met on the surface of HUVEC cells to promote the production of HIF1α, VEGFA, and MMP9, eventually promoting tumor angiogenesis. Previous studies have shown that ESM1 can promote angiogenesis [30,31,32]; however, they only addressed its intracellular function, leaving largely unclear how ESM1, as a secreted protein, acts on endothelial cells to promote angiogenesis. Consequently, we provided new insights into the function of ESM1 during tumorigenesis and progression from the perspective of the interaction between tumor cells and endothelial cells.

**Figure 7:**
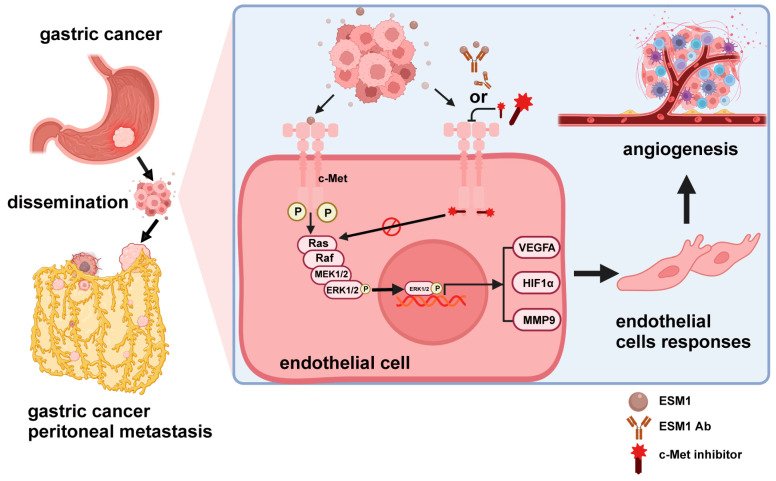
A schematic diagram shows that tumor-derived ESM1 combines with the c-Met expressed by endothelial cells to promote GC peritoneal metastasis by inducing angiogenesis. The function of ESM1 on angiogenesis is through interacting with c-Met, thus activating the MAPK/ERK pathway, which can promote the proliferation, migration, and tube formation of ECs.

The present study found that ESM1 could regulate angiogenesis and promote peritoneal metastasis in gastric cancer. ESM1 was highly expressed in gastric cancer tissues and was significantly correlated with prognosis and MVD. Subsequently, in vitro ESM1 overexpression promoted endothelial cell proliferation, migration, invasion, and tube formation. Mechanistically, ESM1 is secreted by gastric cancer cells to act with the membrane receptor c-Met on endothelial cells, which subsequently activates the MAPK signaling pathway and promotes the expression of pro-angiogenic factors, thereby promoting angiogenesis and peritoneal metastasis (Figure 7).

During peritoneal metastasis, tumor cells shed, remain alive, attach, and invade the peritoneal mesothelial cells and basement membrane, after which they undergo angiogenesis [33]. Angiogenesis during tumor growth and progression is a continuous and dynamic process. Angiogenesis or neovascularization constitutes a compelling therapeutic target considering it is a fundamental process required for the growth and metastasis of many solid tumors [34,35,36], including GC. Dr. Folkman proposed an anti-angiogenesis strategy in 1971, after which many anti-angiogenesis inhibitors targeting different kinds of targets have gradually moved from basic research to clinical application. Previous studies have found that such targeted drugs can down-regulate the expression of vasoactive factors, block tumor neovascularization, promote tumor vascular normalization, and curb tumor growth, recurrence, and metastasis. In addition, they could also improve the tumor immunosuppressive microenvironment and the anti-tumor effect [37,38,39]. For example, bevacizumab inhibits tumor angiogenesis by inhibiting the binding of VEGFA to VEGFR2 [40]. After more than ten years of research and exploration, bevacizumab has opened a new era of personalized tumor treatment. Chemotherapy combined with bevacizumab is the first-line treatment for metastatic CRC which significantly improves the progression-free survival (PFS) of patients [41]. However, bevacizumab has not yet achieved a significant therapeutic effect in gastric cancer-related studies. Moreover, regorafenib has been approved for treating metastatic CRC, metastatic GIST, and second-line drugs in HCC [42,43]. Yet, its long-term efficacy is limited due to drug resistance, highlighting the need for novel antiangiogenic therapeutic targets.

ESM1 is highly expressed in many types of tumors. In addition to promoting the occurrence and development of tumors, its relationship with tumor vascularization also deserves attention. Previous studies revealed that tumors with ESM-1 overexpression were more likely to invade blood vessels in GC, leading to more frequent distant metastases [44]. During cancer invasion and metastasis, MMPs at transcriptional levels can disrupt the surrounding basement membrane, allowing tumor cells to spread into new tissues [45]. It has also been found that ESM1 can regulate MMP9, thereby promoting HCC metastasis and angiogenesis [31], while the specific regulatory mechanism remains to be explored. 

The VEGF pathway is one of the key mediators of angiogenesis in cancer [46]. Also, there is a positive feedback loop between VEGFA and ESM-1. VEGFA stimulates ESM1 expression through the phosphorylation and activation of VEGFR-1. In turn, ESM-1 binds directly to fibronectin, displacing fibronectin-bound VEGFA165, increasing VEGFA bioavailability 165, and subsequently enhancing VEGF-A-mediated signaling [47]. Similarly, HIF1α, as a hypoxia-induced transcription factor, can activate VEGFA transcription [48]. ESM1 can also promote the development of non-small cell lung cancer by regulating the expression of HIF1α [49]. c-Met is a widely expressed cell surface receptor, and existing studies have confirmed that HGF is its classical ligand. The ligands can activate the downstream PI3K/AKT and MAPK pathways and promote the expression of pro-angiogenic factors to induce cell proliferation, migration, invasion, and angiogenesis [50]. In recent years, drugs targeting c-Met have attracted substantial attention, and many drugs targeting c-Met have emerged and achieved results in clinical research [51]. In particular, in 2020, the NDA application for Novartis was accepted by the FDA and granted priority review, while the Fuhong Hanlin anti-c-Met monoclonal antibody entered the clinical trial stage, making the development of c-Met targeted drugs the focus of increasing attention. The c-Met signaling pathway is closely related to the formation and development of gastric cancer [52]. In addition, the effective rate of existing targeted drugs is insufficient, meaning a single drug cannot be used “once and for all”; therefore, combination therapy may have a better effect. Herein, we found that ESM1 may act as a novel ligand of c-Met to promote the expression of downstream pro-angiogenic factors. Accordingly, ESM1 is expected to become a new therapeutic target.

As signaling elements, mitogen-activated protein kinases (MAPK) are essential in translating extracellular stimuli into various cellular responses. Activated in response to environmental stress, inflammatory cytokines, and growth factors, MAPKs regulate downstream targets, including effectors such as transcription factors and pro-angiogenic factors [53]. They induce proliferation, differentiation, survival, migration, angiogenesis, and other carcinogenesis events in gastric cancer cells [54]. The MAPK signaling pathway consists of three branches, namely, the P38 pathway, the ERK pathway, and the JNK pathway. The MAPK cascade consists of three major components, MAPK kinase kinase (MAPKKK), MAPK kinase (MAPKK), and MAPK. Various extracellular and intracellular stimuli, including cytokines, peptide growth factors, hormones, and cellular stressors, can activate the MAPK pathway Herein, we found that ESM1-treated HUVECs could activate the downstream MAPK/ERK signaling pathway, in turn promoting angiogenesis.

In conclusion, ESM1 expression is increased during the peritoneal metastasis of gastric cancer. ESM1 can activate the downstream MAPK/ERK signaling pathway by binding to the c-Met membrane receptor of vascular endothelial cells and can promote the expression of HIF1α, VEGFA, MMP9, and other pro-angiogenic factors, thereby promoting tumor angiogenesis and peritoneal metastasis.

## Data Availability

The data presented in this study are available in this article (and Supplementary Materials).

## Supplementary Materials

The following supporting information can be downloaded at: https://www.mdpi.com/article/10.3390/cancers16010194/s1, Figure S1: ESM1 is up-regulated in primary and metastatic GC tissues and is positively correlated with tumor angiogenesis in GC; Figure S2: ESM1 is up-regulated in GC cells; Figure S3: rESM1 can promote HUVECs proliferation, migration, invasion and tube formation; Figure S4: ESM1 is positively correlated with angiogenesis. Figure S5: Uncropped western blots cited in Figures. Table S1: Primer sequences used in the study; Table S2: Summary of ESM1 expression with clinicopathologic features of gastric cancer.
