# Supplementary material for: ESM1 Interacts with c-Met to Promote Gastric Cancer Peritoneal Metastasis by Inducing Angiogenesis
Source: Cancers (Basel). 2023 Dec 30;16(1):194. doi: 10.3390/cancers16010194 (PMC10778290; doi:10.3390/cancers16010194)
Supplement: Supplementary file 1 [file cancers-16-00194-s001.zip › Supplementary Figures.pdf]

Supplementary data

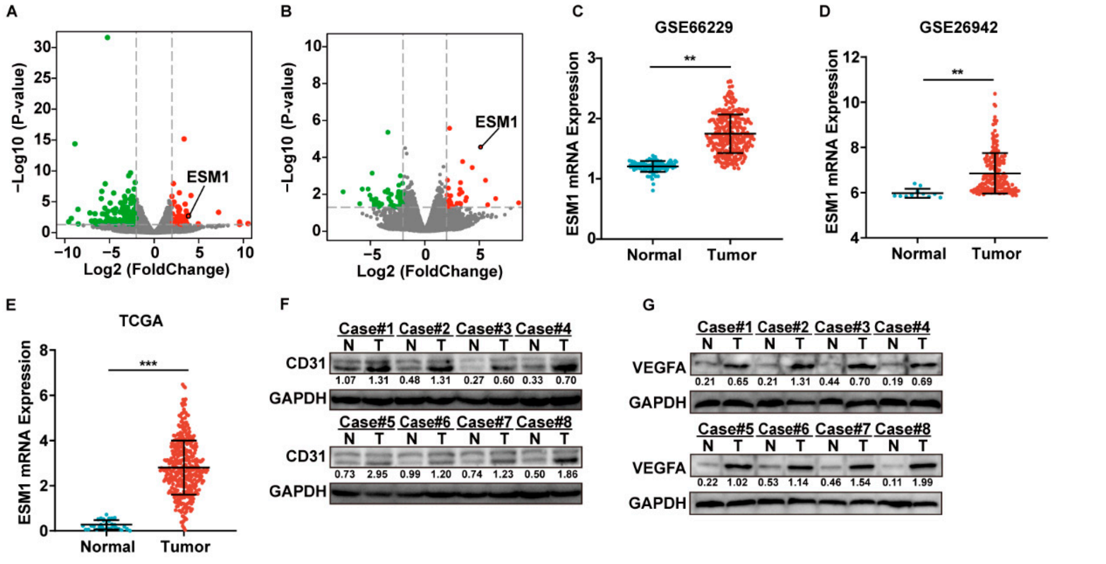

Figure S1. Volcano plots with differentially expressed genes in primary GC **(A)** and metastatic GC **(B)** vs. adjacent normal tissues. **C-E** Higher expression of ESM1 was found in GC samples than the matched normal tissues (based on GSE66229, GSE26942 and TCGA database). **F** and **G** Western blot analysis was performed in 8 pairs of GC patient samples using antibodies against CD31 and VEGFA.

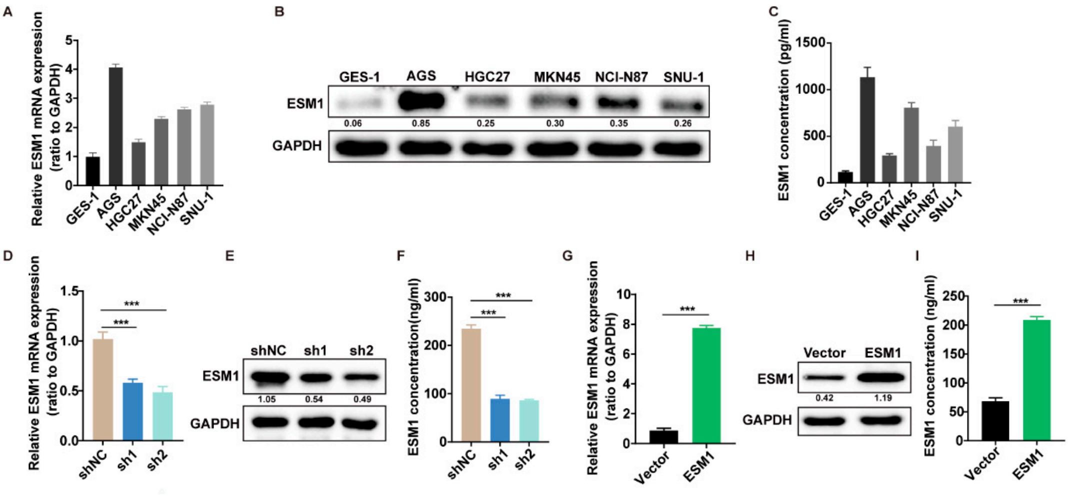

Figure S2. A, B RT-qPCR and Western blot analysis showing the expression of ESM1 in different GC cell lines. Total GAPDH was used as a loading control. **C** ELISA showing the concentration of ESM1 in GC cells supernatant. **D-I** RT-qPCR and Western blot analysis of AGS and HGC27 stably transfected with ESM1 knockdown/overexpression lentiviruses and control lentiviruses. Total GAPDH was used as a loading control.

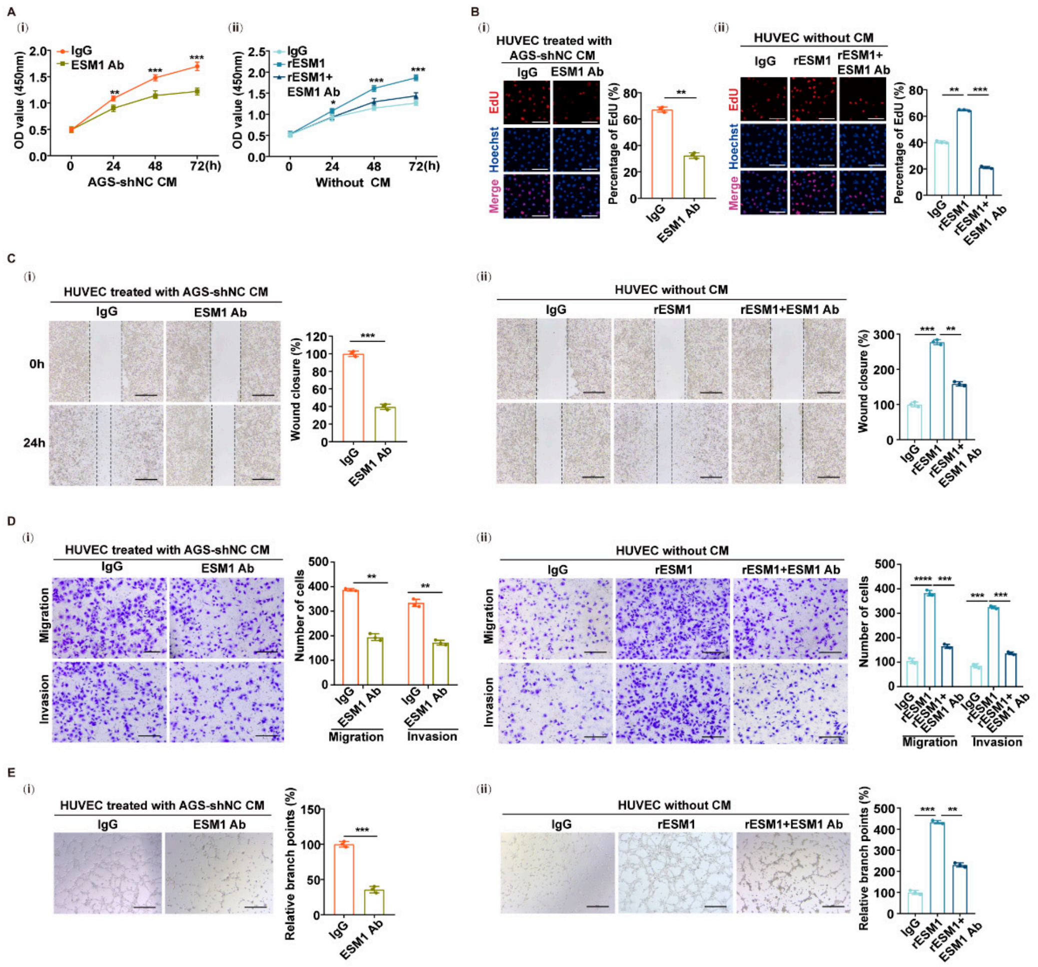

Figure S3. A (i) CCK8 assay analyzed the proliferation of HUVEC treated with CM of AGS-shNC with ESM1 Ab and **(ii)** ECM with recombinant ESM1 or neutralizing antibody against ESM1, and IgG was used as a control. Data are shown as the mean ± SD of triplicate independent sets of experiments; statistical significance was assessed by paired t-test, *<0.05, **<0.01, ***<0.001. **B (i)**EDU assay analyzed the proliferation of HUVEC treated with CM of AGS-shNC with ESM1 Ab and **(ii)** ECM with recombinant ESM1 or neutralizing antibody against ESM1, and IgG was used as a control. Representative images (left panel) and quantification (right panel) are shown as indicated. Data from independent experiments are presented as the mean ± SD. Statistical significance was assessed by an unpaired t-test.

<0.01, ***p < 0.001. Scale bar: 50um. **C (i) Wound healing assay analyzed the migration of HUVEC treated with CM of AGS-shNC with ESM1 Ab and **(ii)** ECM with recombinant ESM1 or neutralizing antibody against ESM1 at 0 and 24h, and IgG was used as a control. Representative images (left panel) and quantification (right panel) are shown as indicated. Data from independent experiments are presented as the mean ± SD. Statistical significance was assessed by an unpaired t-test. **<0.01, ***p < 0.001. Scale bar: 100um. **D** Transwell migration and Matrigel invasion assays were performed to assess migration and invasion ability of HUVEC treated with CM of AGS-shNC with ESM1 Ab and **(ii)** ECM with recombinant ESM1 or neutralizing antibody against ESM1, and IgG was used as a control. Representative images (left panel) and quantification (right panel) are shown as indicated. Data from independent experiments are presented as the mean ± SD. Statistical significance was assessed by an unpaired t-test. **<0.01, ***p < 0.001. Scale bar: 50um. **E** Tube formation assay were performed to assess Tube forming ability of HUVEC treated with CM of AGS-shNC with ESM1 Ab **(i)** and **(ii)** ECM with recombinant ESM1 or neutralizing antibody against ESM1, and IgG was used as a control. Representative images (left panel) and quantification (right panel) are shown as indicated. Data from independent experiments are presented as the mean ± SD. Statistical significance was assessed by an unpaired t-test. **<0.01, ***p < 0.001. Scale bar: 200um.

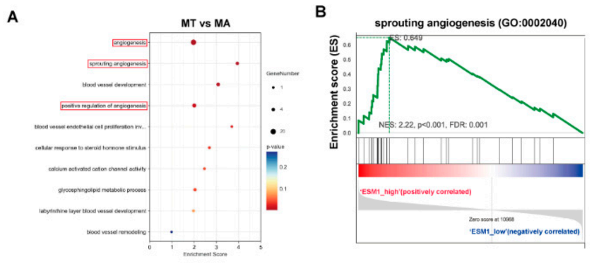

Figure S4. GO **(A)** and GSEA **(B)** analysis of our previous transcriptome sequencing data enriched angiogenesis.

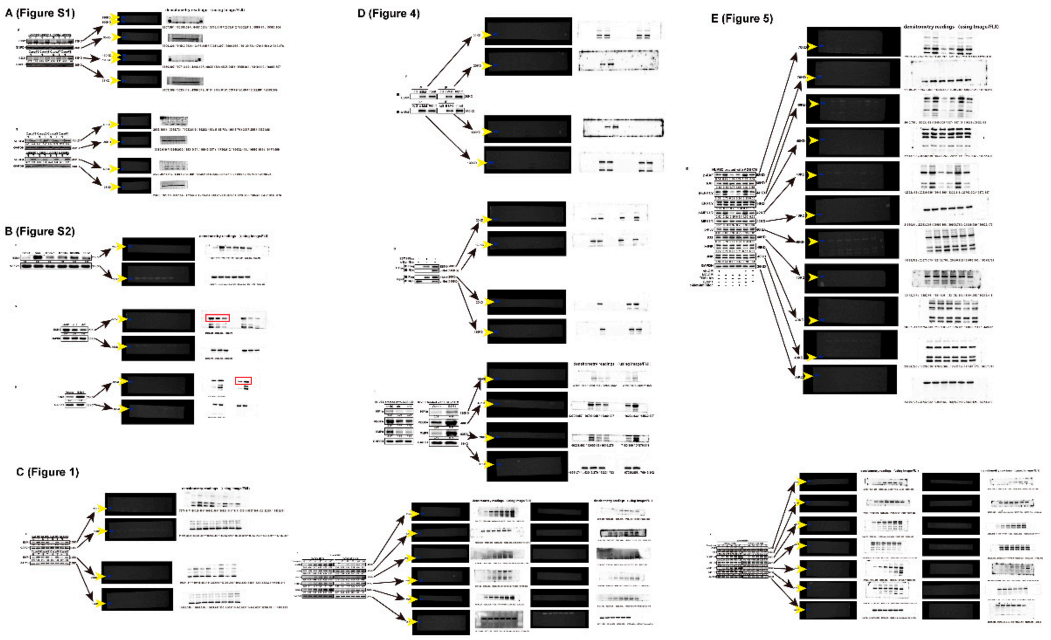

Figure S5. Uncropped western blots cited in Figures.
