# Supplementary material for: ESM1 Interacts with c-Met to Promote Gastric Cancer Peritoneal Metastasis by Inducing Angiogenesis
Source: Cancers (Basel). 2023 Dec 30;16(1):194. doi: 10.3390/cancers16010194 (PMC10778290; doi:10.3390/cancers16010194)
Supplement: Supplementary file 1 [file cancers-16-00194-s001.zip › Supplementary Tables.pdf]

Supplementary Table S1: primer sequences used in the study

ESM1	*F: AGCAATAATTATGCGGTGGACT*<br>*R: ACCAAACTCGTCACCAAAAGGA*
Ang-1	*F: GCAACTGGAGCTGATGGACA*<br>*R: GCTCCCTTTAGTAAAACACCTTCT*
Ang-2	*F: ACTCAGCTAAGGACCCCACT*<br>*R: AGATGCCATTCGTGGTGTGT*
VEGFA	*F: CGGAGCCGCGAGAAGTGCTA*<br>*R: CCTCACCCGTCCATGAGCCC*
FGF2	*F: ATGGCTCCCTTAGCCGAAGT*<br>*R: AGGAAATGCGAACCCACCTG*
MMP9	*F: GGGACGCAGACATCGTCATC*<br>*R: TCGTCATCGTCGAAATGGGC*
MMP1	*F: ATGAAGCAGCCCAGATGTGGAG*<br>*R: TGGTCCACATCTGCTCTTGGCA*
CXCL5	*F: AGCTGCGTTGCGTTTGTTTAC*<br>*R: TGGCGAACACTTGCAGATTAC*
CCL1	*F: ACTGATGTGCCTGCTGCTGG*<br>*R: TGGGGGATCAGGACAGGAGG*
TGFB1	*F: ATGGAGAGAGGACTGCGGAT*<br>*R: TAGTGTTCCCCACTGGTCCC*
IL6	*F: GGAGACTTCACAGAGGATAC*<br>*R: GCATCATCGTTGTTCATACA*
TNF	*F: GAAAGCATGATCCGGGACGTG*<br>*R: GATGGCAGAGAGGAGGTTGAC*
FLT4	*F: TGGAGTTTCTGGCATCTCGT*<br>*R: ACCATCCCACTGTCTGTCTG*
PECAM1	*F: ACATAACAGAGCTGTTTCCCA*<br>*R: AGGACAGGTCCAACAACTC*
VEGFD	*F: ATCTGTATGAACACCAGCACCTC*<br>*R: TGGCAACTTTAACAGGCACTAAT*
IL2	*F: GCAGGATGGAGAATTACAGGAA*<br>*R: GCAGAGGTCCAAGTTCATCTTC*
IL4	*F: TGCCTCCAAGAACACAACTG*<br>*R: GTCCTTCTCATGGTGGCTGT*
EGF	*F: TGTCCACGCAATGTGTCTGAA*<br>*R: CATTATCGGGTGAGGAACAACC*
c-Met	*F: AGCGTCAACAGA GGGACCT*<br>*R: AGCGTCAACAGAGGGACCT*
GAPDH	*F: CTCTGCTCCTCCTGTTCGAC*<br>*R: GCGCCCAATACGACCAAATC*

Supplementary Table S2：Summary of ESM1 expression with clinicopathologic features of gastric cancer

Variables	Cases (n)	ESM1 expression		P Value
		Low 93	High 87	
**Age**				
≤60	61	31	30	0.8707
＞60	119	62	57	
**Gender**				
Male	113	52	51	0.7074
Female	67	41	36	
**Tumor size(cm)**				
＜3.5	91	51	40	0.2106
≥3.5	89	42	47	
**T Stage**				
T1+T2	55	36	19	0.0141*
T3+T4	125	57	68	
**N Stage**				
N0+N1	52	32	20	0.0912
N2+N3	128	61	67	
**M Stage**				
Negative	156	86	70	0.0756
Positive	24	7	17	
**Venous invasion**				
Absence	84	50	34	0.0150*
Presence	106	43	53	
**Distant metastasis**				
Negative	167	93	74	0.0407*
Positive	13	0	13	

*<0.05
